# Application and prospect of intelligent voice technology in the field of agricultural machinery

**DOI:** 10.3389/fpls.2025.1652289

**Published:** 2025-10-27

**Authors:** Xue-ming Xu, Lin Wang, An-qi Zhang, Xiao-fei An, Hao Wang, Xiao-yu Zhu

**Affiliations:** ^1^ School of Broadcast Announcing Arts, Communication University of Zhejiang, Hangzhou, China; ^2^ State Key Laboratory of Intelligent Agricultural Power Equipment, Beijing, China; ^3^ Intelligent Equipment Research Center, Beijing Academy of Agriculture and Forestry Sciences, Beijing, China; ^4^ Nongxin Technology(Beijing)Co., Ltd, Beijing, China

**Keywords:** intelligent voice, agricultural machinery, voice control, fault warning, voice service

## Abstract

With the development of smart agricultural machinery technology, the application of intelligent voice technology in smart agricultural machinery is becoming increasingly widespread. As a new interactive method, intelligent voice technology can reduce operator workload, improve operational quality, and enhance operational safety in agricultural machinery. This article reviews the current state of development of intelligent voice technology in agriculture, summarizing the bottlenecks and challenges in its application within the agricultural machinery field. It further proposes a framework for intelligent voice technology in agricultural machinery and presents technical implementation plans for two typical application scenarios: intelligent control and fault diagnosis/early warning of agricultural machinery. These findings aim to provide a reference for the application of intelligent voice technology in the agricultural machinery sector. Finally, development suggestions are proposed, including building specialized voice recognition vocabularies and semantic parsing models, exploring the application of artificial intelligence in agricultural machinery voice technology, and establishing relevant technical standards for intelligent voice applications in agricultural machinery. This article provides important references and insightful ideas for the development of intelligent voice technology in the agricultural machinery field, which will facilitate the promotion and adoption of intelligent agricultural machinery technologies.

## 1 Introduction

With the continuous advancement of the intelligence level of agricultural machinery, various new control and interaction methods, such as intelligent screens, electronic control handles, and control panels, have become increasingly widespread. On the one hand, the application of these new interaction methods addresses the shortcomings of traditional agricultural machinery, such as limited interaction pathways and inconvenient mechanical operation. On the other hand, it also increases the difficulty of use for users to a certain extent (Changyuan et al., 2022a; Chuang., 2022; Zhanhong et al., 2024). For example, in an agricultural machinery automatic navigation system, users need to set navigation parameters and operate application software interfaces. This often leaves agricultural machinery operators confused and leads to a poor user experience. Therefore, developing a new intelligent interaction method for agricultural machinery would be beneficial. It aims to reduce the learning and operational complexity of systems like automatic navigation and precision control for seeding, fertilization, and pesticide application. This would encourage the dissemination and adoption of intelligent agricultural machinery technologies. It would also facilitate the transformation and upgrading of agricultural machinery toward higher levels of intelligence.

At present, various new interaction technologies, such as voice interaction, gesture interaction, and brain-computer interfaces, are developing rapidly. Among them, intelligent voice technology has achieved relatively mature development and application, serving as a typical example of technological breakthroughs in the era of digital intelligent media. The development of voice technology has undergone a long journey. Early speech recognition technology could only enable machines to respond to a limited number of sounds, with a relatively low level of intelligence. With the application of technologies such as computers and artificial intelligence in the field of speech recognition, intelligent voice technology has gradually developed and improved (Xiaoyan et al., 2001; Huanliang et al., 2006; Fumei and Yu, 2023). The origins of intelligent voice technology can be traced back to 1952. That year, Bell Labs developed the Audreyi system, which could recognize Arabic numerals. In the 1980s, the Hidden Markov Model (HMM) and Artificial Neural Networks (ANN) became widely applied in speech recognition. These advancements significantly improved speech recognition performance. Subsequently, with the support of AI technologies such as deep learning, end-to-end models, natural language processing, and semantic understanding (Suifang et al., 2005; Yexin, 2024; Zenghui et al., 2024), intelligent voice technology began achieving recognition capabilities approaching human levels. Subsequently, with the support of AI technologies such as deep learning, end-to-end models, natural language processing, and semantic understanding, intelligent voice technology advanced rapidly. It began achieving recognition capabilities that approach human levels. Intelligent voice interaction has gradually evolved from “hearing clearly” to “understanding accurately.” It has transitioned from laboratories to everyday life, with applications such as Siri, Xiao Ai, Xiaoyi, and Xiaobu becoming representative examples of this technology (Shuguang, 2007; Ling, 2023). Digital voice recognition and speech synthesis have expanded from purely meeting communication needs to becoming a crucial method of human-computer interaction. This has significantly reduced the challenges faced by the elderly, children, and individuals with disabilities in connecting and interacting with the external world, while also improving the overall interaction experience (Rabiner and Juang, 1986; Juang and Rabiner, 2005; Ghai and Singh, 2012; Yu and Deng, 2016). iFlytek has developed the Spark Cognitive Model, which possesses advanced capabilities in intelligent voice interaction, intelligent question answering, logical reasoning, and analysis. In the domain of voice interaction, it has achieved breakthroughs in multi-language speech recognition, synthesis, and translation technologies, supporting seamless dialogues across 74 languages and dialects without manual switching, thereby enabling smooth and natural voice input and output across various scenarios. In blind tests comparing with international vendors, the model demonstrates parity across 60 languages and leads in 14 key languages. iFlytek’s intelligent in-car voice system supports 23 major languages and is integrated into vehicles sold in over 60 countries and regions. Similarly, the notable achievements of Speech Ocean include ultra-natural speech synthesis technology and a full-chain intelligent dialogue system. These advancements have overcome critical challenges in acoustic signal processing, speech recognition and synthesis, as well as natural language understanding. By employing speech feature discretization technology and large language models, the difficulty of predicting continuous speech features has been effectively reduced. In practical applications, these technologies have been deployed across industries such as banking, government services, and transportation, offering solutions such as intelligent customer service and smart voice-based ticketing systems, thereby driving the intelligent transformation and upgrading of various sectors (Yiqun et al., 2024; Jiang et al., 2024; Xinhe et al., 2025; Jovan et al., 2024; Houan et al., 2024).

As a new form of human-computer interaction, intelligent voice technology can parse and understand the voice commands of agricultural machinery operators, integrating with intelligent control systems of agricultural machinery. This enables the automatic configuration of operating parameters and the control of intelligent application software. By accurately translating the operator’s intent into machine actions, it effectively meets the needs of agricultural production. Therefore, intelligent voice technology has the capability to transform the current methods of agricultural machinery operation, control, maintenance, and service, showcasing significant application potential. Currently, many countries, including China, have systematically deployed industrial layouts for intelligent voice technology in areas such as communication calls, information query systems, text processing, home automation, vehicle and aircraft navigation, and driving. In the field of agricultural machinery and equipment, it is urgently necessary to conduct an in-depth and systematic review and summary of intelligent voice technology. This includes exploring the technical and developmental pathways for improving the interactive capabilities and application experience of modern agricultural machinery. It also involves applying this technology to the agricultural machinery sector as quickly as possible.

This article reviews the current state of development of intelligent voice technology in the agricultural sector, summarizing the bottlenecks and challenges that remain in its application within the field of agricultural machinery. Subsequently, it elaborates on the framework of intelligent voice technology for agricultural machinery and proposes targeted technical solutions for two typical application scenarios: intelligent control of agricultural machinery and fault early warning and diagnosis. Finally, the article provides an outlook on the future development prospects of intelligent voice technology in the agricultural machinery domain and offers recommendations for its advancement.

## 2 The development status of voice technology in the agricultural sector

With the development of information technology, intelligent voice technology has gradually expanded into the agricultural sector. As a labor-intensive industry, agriculture can greatly benefit from the application of intelligent voice technology, which helps to significantly reduce labor efforts and improve agricultural production efficiency. At present, voice technology is widely applied in areas such as agricultural UAVS, livestock and poultry farming, and agricultural technical Q&A. These applications aim to enhance agricultural productivity, precision, and sustainability while addressing the issue of labor shortages in the agricultural sector.

### 2.1 The application of intelligent voice technology in the field of agricultural UAVs

As a new type of agricultural machinery, agricultural UAVs hold significant value in field crop agriculture. During operations, UAVs require interaction with operators, and advanced intelligent voice technology can enhance human-machine interaction, optimize the operational experience, and further improve the quality and efficiency of UAV operations (Ghai and Singh, 2012). During the operation of UAVs, challenges such as wind noise, human voices, and other environmental sound interferences, as well as low recognition rates for these noises, are prevalent. Therefore, current research primarily focuses on improving the accuracy of UAV voice recognition. Modern speech recognition typically employs acoustic models based on recurrent neural networks (RNN). Ravanelli et al (Ravanelli et al., 2018). modified one of the RNN models, the gated recurrent unit (GRU), and proposed a more simplified architecture known as lightweight GRU. This modification not only reduces the training time per iteration but also adapts to a wider range of tasks, input features, noise conditions, and spans across various speech recognition paradigms. For example, Pan et al (Jiaqi et al., 2021). proposed a noise reduction solution by designing a system that integrates the Wiener filtering noise reduction algorithm and WebRTC technology, effectively addressing the noise reduction challenges in UAV voice systems and enabling audio-visual interaction. In the field of UAV control technology, some researchers have achieved multimodal interaction between humans and UAV swarms by integrating voice and gesture models and constructing a dual autonomous recognition framework (Jingxing et al., 2021; Yingke et al., 2021; Mingrui, 2024).

### 2.2 The application of voice technology in the field of livestock and poultry farming production

In livestock and poultry farming production, intelligent voice technology is typically used for monitoring or interpreting the physiological conditions and behavioral characteristics of livestock and poultry. Through intelligent audio analysis, it can be discovered that the sounds emitted by animals contain multiple layers of information. As an important branch of intelligent voice technology, intelligent sound monitoring technology holds significant potential for application in the field of livestock and poultry farming. At present, domestic and international scholars have conducted systematic and in-depth research by introducing models and algorithms such as deep learning in areas such as goat cough sound recognition (Chuanzhong, 2016), poultry emotion and pathology recognition (Ligen et al., 2013; Lee et al., 2015; Banakar et al., 2016), swine pathology recognition (Chung et al., 2013), and cattle behavior state recognition (Clapham et al., 2011). For example, Zhong et al (Jiaqi et al., 2021). used an improved recognition model (HMM) to identify goat cough sounds, achieving a recognition accuracy of 92.5%. LEE et al (Lee et al., 2015). developed a poultry stress response monitoring system, achieving a recognition accuracy of up to 96.5% in the identification and analysis of broiler chicken vocalizations under specific conditions. BANAKAR et al (Banakar et al., 2016). designed an intelligent audio device using data mining and D-S evidence theory, which can diagnose diseases such as Newcastle disease, bronchitis, and avian influenza in chickens by decomposing and analyzing portions of their sound signals. The aforementioned technological research provides strong support for intelligent prevention and control technologies in livestock and poultry farming. It enables the early warning and diagnosis of livestock and poultry diseases, helping to reduce production losses in farming.

### 2.3 Application of intelligent voice technology in the field of agricultural technology service.

In the field of agricultural technology services, agricultural intelligent question-and-answer systems can provide convenient information services for agricultural practitioners. Currently, these systems are widely applied in areas such as seed cultivation, pest and disease control, livestock and poultry disease prevention, and crop planting. They offer timely and professional solutions to farmers’ inquiries, significantly improving agricultural production efficiency. Currently, many scholars develop related intelligent question-and-answer systems based on the principles of knowledge graphs. LI, Zhang, Zhou, and others (Zihao, 2021; Mingmei, 2022; Li, 2023) have developed intelligent Question and Answer (Q&A) systems focusing on agricultural cultivation techniques, as well as kiwifruit and tea planting. Xia, Zhu and others (Yingchun, 2018; Tingting, 2023) developed a crop pest and disease Q&A system based on an agricultural pest and disease knowledge graph as the foundational knowledge base. Zhang et al (Pengpeng, 2023). designed and developed a professional knowledge Q&A system in the field of dairy cow diseases based on a knowledge graph. The system provides convenient and efficient Q&A services for dairy cow farmers, making the dairy farming process more modernized and intelligent. Pang, Si, and others (Hejie, 2023; Wenting, 2023) designed and developed an intelligent Q&A system for wheat and corn breeding, enabling knowledge-based question-and-answer interactions in the field of wheat and corn breeding.

### 2.4 Summary and bottleneck analysis

Intelligent voice technology is increasingly being applied to various aspects of agriculture, providing new technical means and support for the development of smart agriculture. However, due to the unique production scenarios in agriculture, intelligent voice technology also faces certain bottlenecks and challenges.

#### 2.4.1 Environmental noise and robustness of speech recognition

Agricultural work environments are complex and variable, particularly during field machinery operations. Background noises such as engine sounds, wind noise, and mechanical vibrations create significant interference, severely affecting the accuracy and robustness of speech recognition systems. Existing speech recognition models are often trained in quiet environments, making them less adaptable to high-noise conditions. As a result, they are prone to misrecognition or recognition failures in noisy agricultural scenarios. In addition, the acoustic characteristics of different operational scenarios (such as dry fields, paddy fields, and greenhouses) vary significantly, making it challenging for a single model to generalize and adapt effectively across diverse environments.

#### 2.4.2 Real-time performance and computational resource constraints

The operation of agricultural machinery has extremely high requirements for system real-time performance, and the recognition and response to voice commands must be completed within a very short time; otherwise, it may affect operational safety and efficiency. However, high-precision speech recognition models often have high computational complexity and strict requirements for hardware processing power. The current computational capabilities of agricultural machinery terminal devices are limited, making it difficult to deploy complex deep learning models. How to achieve low-latency and high-accuracy speech processing with limited computational resources is a significant technical challenge.

#### 2.4.3 Insufficient semantic understanding and contextual association capabilities

Agricultural scenarios involve a large number of specialized terms and context-related commands (such as “increase the rotation speed” or “switch to seeding mode”). Current voice systems still show limitations in semantic understanding and multi-turn dialogue capabilities. Especially in unstructured natural language interactions, the system is prone to misunderstand user intentions, leading to control errors or inaccurate responses. Enhancing the model’s contextual awareness and domain adaptability is a key focus for the future.

#### 2.4.4 Data scarcity and high annotation costs

The collection and annotation of agricultural voice data face practical challenges: ① Agricultural scenarios are diverse, and data distribution is uneven. ② Annotating voice data requires specialized knowledge, leading to high costs. ③ Privacy concerns and regional differences hinder data-sharing mechanisms, which remain underdeveloped.

The lack of high-quality, large-scale, and well-annotated agricultural voice datasets severely limits model training and optimization.

#### 2.4.5 Ensuring security and reliability

The operational environment of agricultural machinery is complex. If the voice system experiences misrecognition or response delays, it could potentially lead to safety accidents. The stability and fault tolerance of current voice systems under extreme conditions still need improvement. Designing a voice control system with mechanisms such as fault self-diagnosis, command verification, and emergency switching is key to ensuring the safety of agricultural machinery operations.

#### 2.4.6 The lack of standardization and system compatibility

Currently, agricultural intelligent voice systems lack unified technical standards and interface specifications. Equipment from different manufacturers often struggles to interconnect, with discrepancies in voice command sets, communication protocols, and data formats. This limits the scalability of technology promotion and the development of an application ecosystem.

As agricultural machinery moves toward greater intelligence and informatization, intelligent voice technology, with its unique technical advantages, will gradually integrate into smart agricultural machinery. The technical solutions for intelligent voice technology in the aforementioned application scenarios essentially revolve around two key aspects: voice recognition and voice control. However, unlike typical application scenarios such as agricultural drones, livestock farming, and agricultural Q&A systems, intelligent voice technology in the agricultural machinery field has its own particularities and independence. Moreover, agricultural machinery operating environments are more complex and demand higher levels of real-time performance, safety, and reliability. Therefore, this article will focus on elaborating the intelligent voice technology system for agricultural machinery and its typical application scenarios, providing reference ideas for the application and development of intelligent voice technology in the field of smart agricultural equipment.

## 3 Agricultural machinery intelligent voice technology system and implementation plan

### 3.1 Agricultural machinery intelligent voice technology system

In-vehicle voice services are currently developing rapidly in the automotive field, and artificial intelligence technology has made it easy to enable voice interaction between drivers and vehicles. Modern intelligent car cockpits utilize technologies such as voice recognition, speech-to-text conversion, semantic analysis, and natural language understanding. Centered on human-computer interaction in in-vehicle scenarios, they integrate intelligent navigation, multimedia entertainment, vehicle control, driving behavior monitoring, and vehicle condition monitoring to meet the human-computer interaction needs of smart cockpits. By focusing on “voice interaction intelligence + cloud-connected services,” they transform the traditional control model of in-vehicle devices, which relied on touch and buttons, enhance the interaction experience, and effectively ensure driving safety. Regardless of how automotive intelligence technology evolves, safety, convenience, and comfort remain the core goals of cockpit design. Centered around these three goals, intelligent automotive cockpits have been widely implemented. Starting from early applications like multimedia entertainment, intelligent navigation, and vehicle condition monitoring, intelligent automotive cockpits have now evolved to include control of some core vehicle functions, driving behavior monitoring, and correction.

**Figure 1:**
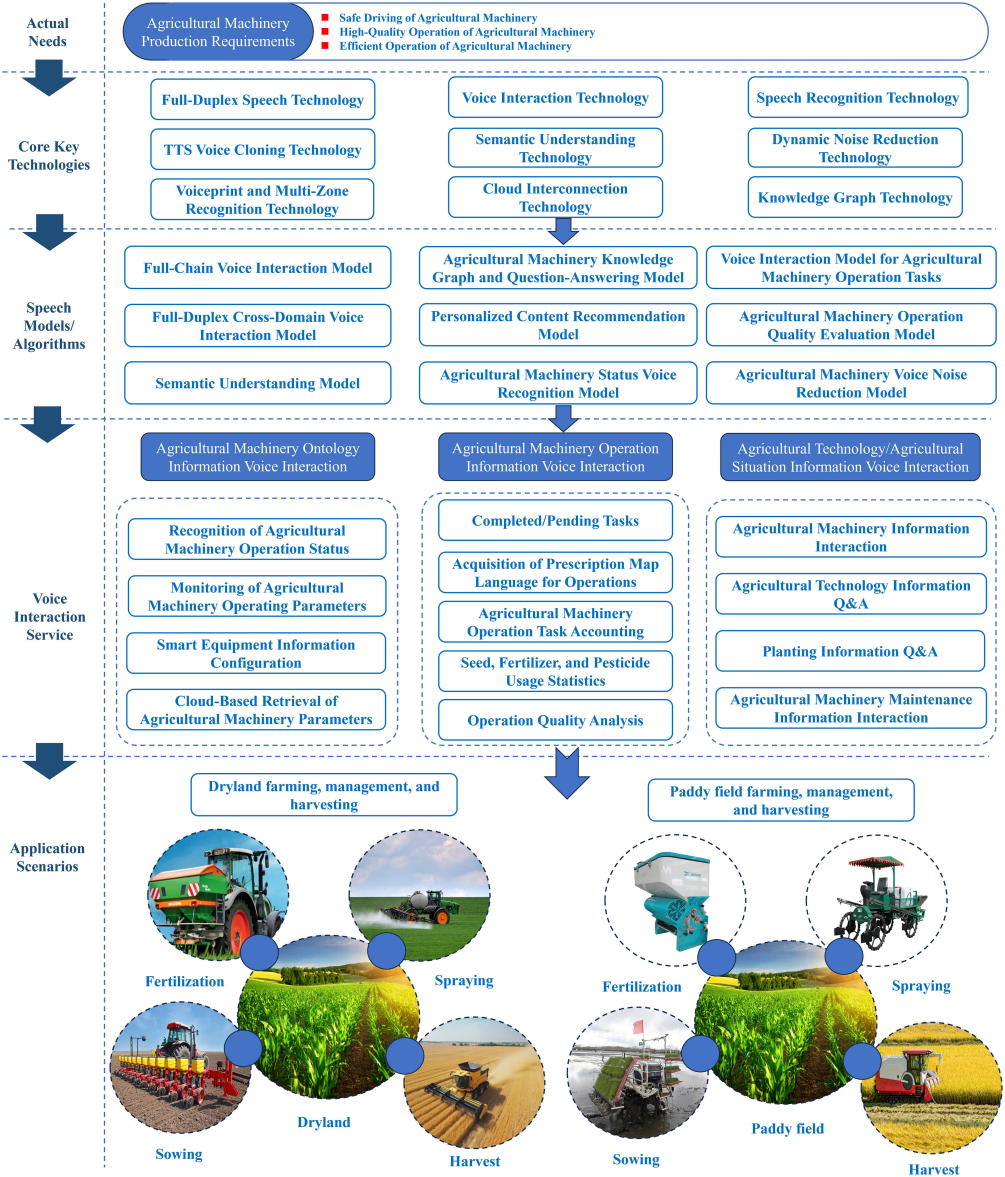
Implementation plan for intelligent voice service of agricultural machinery.

Currently, agricultural production in China is still primarily mechanized, with Agriculture 1.0, 2.0, 3.0, and 4.0 developing in parallel. The future trend is toward intelligentization. Compared to voice interaction services in intelligent automotive cockpits, certain key technologies are shared, such as full-duplex voice and voice cloning technologies. However, considering the actual conditions of Chinese agricultural machinery and agronomy, more critical technologies need to be overcome to achieve multimodal interaction centered around voice. At present, the core needs of agricultural machinery operations remain safety, high quality, and efficiency. Addressing these practical requirements for intelligent voice technology in agricultural machinery, the system of agricultural machinery intelligent voice technology encompasses three categories: voice interaction services for agricultural machinery body information, voice interaction services for agricultural technology/agricultural situation knowledge graphs, and voice interaction services for agricultural machinery operation information, as illustrated in **Figure 1**.

In terms of voice interaction services for agricultural machinery body information, technologies such as deep learning, dynamic noise reduction, and semantic understanding are employed to establish models like agricultural machinery voice noise reduction and machinery state recognition. These provide services such as operational status recognition, monitoring of machinery operating parameters, intelligent equipment parameter settings, and cloud-based retrieval of machinery parameters. The system supports multi-turn dialogue capabilities based on data and knowledge, domain-specific error correction dialogue services, and voice reminders to ensure the safe operation of agricultural machinery.

In terms of voice interaction services for agricultural technology and agricultural situation knowledge graphs, technologies such as voiceprint and multi-accent recognition, knowledge graphs, and heuristic question-answering are utilized. By integrating a built-in knowledge base, models like agricultural situation knowledge graph Q&A and personalized content recommendation are established. These enable services such as agricultural situation information interaction, agricultural technology information delivery, and agricultural machinery maintenance information interaction. Based on this information, precise agricultural machinery operations and scheduling can be achieved.

In terms of voice interaction services for agricultural machinery operation information, technologies such as voice recognition, dynamic noise reduction, and cloud connectivity are employed to establish models like the agricultural machinery operation task voice interaction model and the operation quality evaluation model. These provide services such as remote retrieval of navigation lines, voice access to operational prescription maps, real-time interaction for completed and pending tasks, intelligent calculation of operation tasks, voice calling for agricultural supplies, statistics on seed, fertilizer, and pesticide usage, and operation quality analysis. These services deliver specialized and efficient voice support for all stages of farming operations, including plowing, planting, management, and harvesting in both paddy and dry fields.

### 3.2 Implementation plan for intelligent voice control technology in agricultural machinery

**Figure 2:**
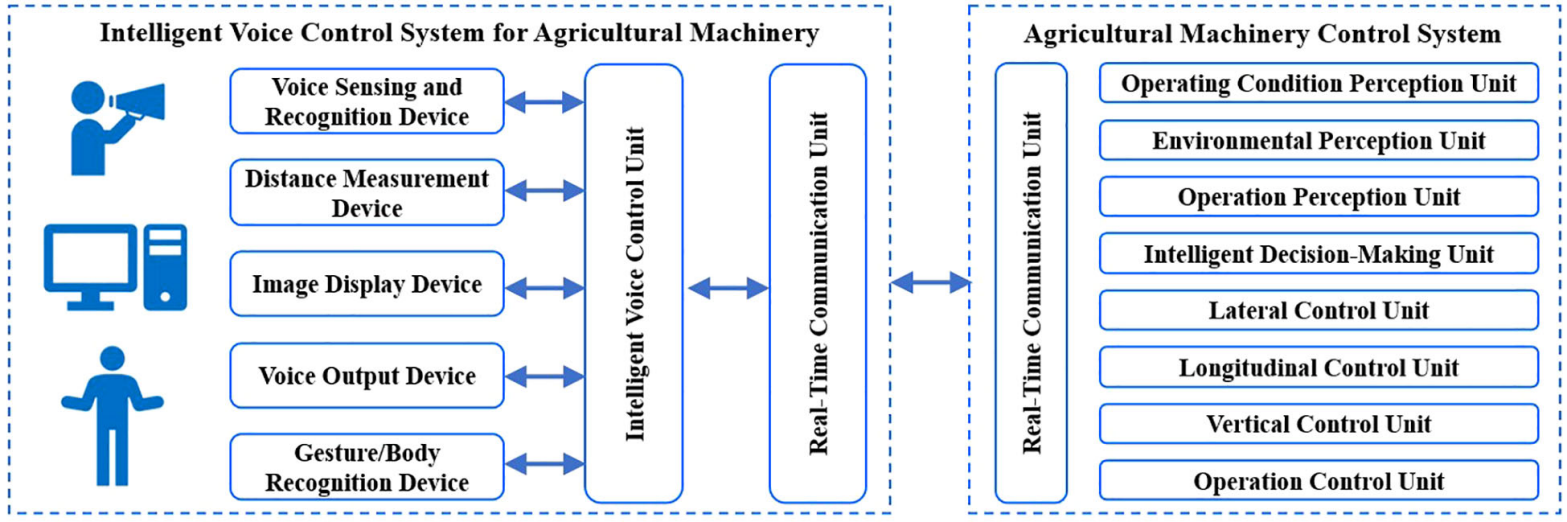
Typical scheme of intelligent voice control system for agricultural machinery.

Controlling agricultural machinery through a remote controller has the major disadvantage of being inconvenient to carry and operate. Voice-based human-machine interaction is the simplest approach, freeing the operator’s hands and enabling them to perform other tasks simultaneously. In the complex working environment of agricultural machinery operations, the intelligent voice control system must meet the requirements of high real-time performance, high accuracy, and high reliability. When intelligent agricultural machinery performs tasks through voice control, any misrecognition of a voice command could potentially lead to major accidents. A typical intelligent voice control system for agricultural machinery generally consists of functional modules such as voice perception and recognition, distance measurement, voice output, and image display, as well as an intelligent voice control unit and a real-time communication unit (Wanbing et al., 2021), as shown in **Figure 2**. To enhance human-machine friendliness, the system can also be equipped with gesture/body recognition modules for operators (Lingfei et al., 2022).

The voice perception and recognition device consist of a microphone array and a voice recognition module, providing voice perception and recognition functions. By integrating information from the distance measurement device with the voice perception and recognition device, the operator’s position can be determined (Yukun et al., 2022). The intelligent voice control system for agricultural machinery automatically adjusts the parameters of the voice perception and recognition device based on the operator’s distance and position relative to the vehicle, thereby improving the sensitivity and accuracy of voice recognition.

The intelligent voice control unit is the core of the agricultural machinery intelligent voice control system. It includes modules such as the voice command library, command encoding, intelligent decision-making, and system parameter configuration. The intelligent voice control unit matches the recognized commands with the command library, maps them into corresponding command codes, and outputs them to devices such as image display, voice output, and the real-time communication unit. The execution results of the voice control commands for the agricultural machinery control system, along with information on the machinery’s operating status, environment, and tasks, are output to devices such as image displays and voice output units.

The real-time communication unit may include CAN bus, radio, Bluetooth, and WLAN communication methods. It maps the intelligent voice control unit into corresponding command codes and sends them to the agricultural machinery control system following the respective communication protocol standards. The execution results of the agricultural machinery control system’s voice control commands, along with information on the machinery’s operating status, environment, and tasks, are fed back to the agricultural machinery intelligent voice control system through the real-time communication unit.

Taking tractors and combine harvesters as examples of typical agricultural machinery, intelligent voice technology can be utilized during tractor operations to enable real-time adjustments to the tractor’s start, stop, and operating speed. Additionally, it can regulate the operational parameters of attached implements, such as plowing depth, seeding rate, fertilization rate, and pesticide application rate. During field transportation, intelligent voice technology can facilitate functions such as cruise control and automatic obstacle avoidance, significantly reducing the operator’s workload and driving intensity. In harvesting scenarios, intelligent voice technology can enable real-time control of the header height, operating speed, reel speed, and fan speed. Furthermore, when the harvester operator receives a voice alert indicating that the grain tank is full, the “start unloading” voice command can be used to initiate automatic grain unloading, significantly freeing the operator’s hands. In the future, intelligent voice control systems for agricultural machinery should support multilingual recognition and ensure accurate voice recognition in noisy field environments. This would minimize the occurrence of errors caused by misrecognition of voice commands, thereby enhancing operational efficiency and safety.

### 3.3 Implementation plan for agri-machinery fault diagnosis and early warning

The intelligent voice research related to agricultural machinery fault diagnosis and warning primarily focuses on self-propelled power systems such as tractors and harvesters (Zhijun et al., 2023), as well as intelligent agricultural equipment such as seeders and plant protection machines (Xiwen et al., 2022). It involves data analysis, fault diagnosis, and voice warnings for the fatigue reliability and functional reliability of the entire machinery, supporting implements, and key components under typical high-speed and heavy-load operating conditions (Lizhang et al., 2014; Changkai et al., 2019; Changkai et al., 2022; Yanxin et al., 2022b; Changkai et al., 2023).

**Figure 3:**
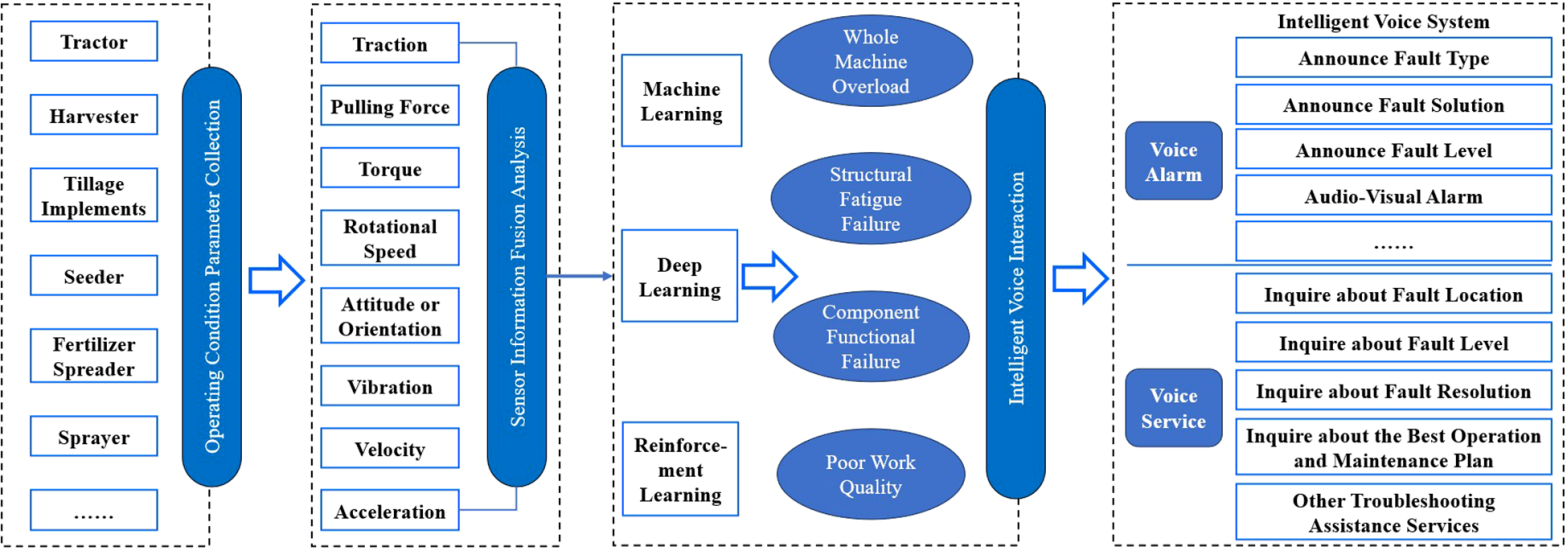
Architecture of intelligent voice warning technology for agricultural machinery fault diagnosis.

The intelligent voice warning technology for agricultural machinery fault diagnosis takes intelligent agricultural machinery as its application target (Xiwen et al., 2021; Yanxin et al., 2022a) and is supported by data and information. It deeply integrates modern technologies such as new materials, intelligent sensing, intelligent measurement and control, the Internet of Things, big data, cloud computing, and artificial intelligence with agricultural machinery. This enables functions such as operating condition data collection, real-time data uploading, massive data storage, key data integration, fault diagnosis and decision-making, and intelligent voice prompts for agricultural machinery during actual production operations (Chengliang et al., 2020; Xuegeng et al., 2020; Changyuan et al., 2022b; Xiwen et al., 2024). The technology guides intelligent agricultural machinery to efficiently and effectively complete various agricultural production activities, including plowing, planting, management, and harvesting. The technical architecture of the intelligent voice warning for agricultural machinery fault diagnosis is shown in **Figure 3**.

The intelligent voice warning technology for agricultural machinery fault diagnosis selects key operational parameter measurement data and develops a dynamic data acquisition system for multi-operational load and condition parameters of agricultural machinery. Field operation measurement data are obtained through methods such as CAN bus, serial communication, analog data acquisition, and digital data acquisition. These data are transmitted in real-time to the onboard controller and uploaded for storage in a cloud database. The system integrates machine learning algorithms such as support vector machines, clustering, decision trees, Bayesian models, and gradient boosting. It also incorporates deep learning algorithms such as convolutional neural networks, graph convolutional neural networks, long short-term memory networks, generative adversarial networks, and reinforcement learning. This enables the formation of fault diagnosis decision categories, including whole-machine overload, component functional failure, structural fatigue failure, poor operational quality, and insufficient operational efficiency. Furthermore, intelligent voice warning settings and development are implemented through fault severity classification, warning method combination, warning decibel grading, warning frequency adaptation, and warning location selection. Ultimately, this results in the creation of intelligent voice warning technology and systems for fault diagnosis that can be applied to various agricultural machinery and operational conditions.

The intelligent voice warning technology for tractor fault diagnosis can identify hazardous scenarios in real-time, such as engine overheating and overloading, excessive machine sinking and slippage, component fatigue failure, and three-point hitch breakage. This technology effectively prevents various extreme operating conditions, including prolonged tractor overloading, high fuel consumption, and low-efficiency operations. The intelligent voice warning technology for combine harvester fault diagnosis can analyze the overall power performance, economic performance, and operational status of key components. It enables effective warnings for hazardous situations such as header grounding, drum blockage, feeder blockage, and excessive harvester vibration. And the intelligent voice warning technology for fault diagnosis of agricultural implements can accurately identify inefficient, low-quality, or even hazardous operating conditions of typical machinery. These include seeders missing or double-seeding, fertilizer spreaders under-fertilizing or over-fertilizing, plow body-frame separation in tillage equipment, and uneven spraying by plant protection machines. This ensures the efficient, accurate, and safe completion of critical agricultural field operations.

Therefore, the practical application of intelligent voice technology in agricultural machinery fault diagnosis and warning is highly necessary. By accurately integrating new technologies such as advanced materials, intelligent sensing, intelligent measurement and control, the Internet of Things, big data, cloud computing, and artificial intelligence, it is possible to achieve intelligent voice warnings for extreme loads, hazardous conditions, and potential faults under high-speed and heavy-load operations. This holds significant importance for improving the operational reliability and functional reliability of intelligent agricultural machinery.

## 4 Future development trends and recommendations

Intelligent voice technology has become one of the key methods for human-computer interaction. The integration of digital intelligent voice technology with agricultural machinery is a gradual process, reflecting both the general pattern of technological development and the need for humans to adapt to technological changes. In the future, with continuous technological advancements and the development of the smart agricultural machinery industry, intelligent voice technology will bring more convenience and smarter experiences to farmers’ production and daily lives.

### 4.1 Future development trends

Intelligent voice technology is highly adaptable and widely used, with large-scale applications in fields such as automobiles and airplanes. Since the operation of agricultural machinery is similar to that of automobiles, intelligent voice technology also shows great potential in the agricultural machinery field. Based on the characteristics of agricultural machinery operations, intelligent voice technology is expected to be applied in areas such as machinery operation and management, intelligent decision-making support, voice alerts and safety reminders, as well as user training and support.

#### 4.1.1 Agricultural machinery operation and management

Farmers can use voice commands to control the operational tasks and task assignments of equipment such as tractors and combine harvesters. This reduces complex or repetitive manual operations while enabling remote monitoring of agricultural machinery. It also improves operational efficiency for large-scale farms and in remote areas.

#### 4.1.2 Intelligent decision-making support

Agricultural machinery can analyze collected farmland and crop data to generate and deliver voice reports on crop growth conditions, soil status, and other analyses, assisting farmers in making informed decisions.

#### 4.1.3 Voice alerts and safety reminders

Agricultural machinery can automatically diagnose equipment issues and provide repair suggestions, real-time operational safety reminders, and emergency alerts through intelligent voice technology. This helps farmers avoid potential dangers, reduce downtime, and lower maintenance costs.

#### 4.1.4 User training and support

Agricultural machinery can provide operation tutorials and training through intelligent voice assistants, helping new users quickly get started. Additionally, when users encounter operational issues, they can interact with the support system via voice to receive instant help and solutions.

In the short term, intelligent voice technology requires a process of familiarization and adaptation. However, the agricultural machinery industry urgently demands automation, intelligence, and efficiency. Current smart agricultural machinery faces technical challenges and market needs. Therefore, intelligent voice technology is expected to have broad application prospects in this field.

### 4.2 Development suggestions

The complex and variable operating environment of agricultural machinery, along with differences in agricultural production models and user proficiency across regions, pose significant challenges to the application of intelligent voice technology in the agricultural machinery field. Considering the vast territory, diverse ethnic groups, and complex dialects in China, intelligent voice systems should be capable of recognizing multiple languages and dialects. In agricultural machinery, the multilingual and dialect recognition function will greatly enhance the system’s universality and usability. Therefore, it is recommended to conduct in-depth research on the application of intelligent voice technology in the agricultural machinery industry from the following three aspects:

(1) Build a specialized vocabulary database and semantic parsing model for agricultural machinery.

The core application of intelligent voice recognition and control systems in agricultural machinery is to convert operators’ voice commands into control signals for precise machinery operation. This requires efficient and accurate voice recognition algorithms and optimized control architectures to ensure real-time and accurate command execution. Due to the diversity of dialects and accents among agricultural workers, traditional systems based on standard Mandarin face significant challenges. Therefore, it is crucial to develop voice recognition models compatible with multiple dialects and adaptable to accent variations or provide user-customized training functions. General systems have a low recognition rate for agricultural terms, crop names, and machinery components. This makes it necessary to create specialized agricultural vocabulary and semantic models. These models ensure accurate command interpretation and precise control.

(2) Explore the application of large models and generative AI in agricultural machinery intelligent voice systems.

In agricultural machinery, voice prompt and interaction systems provide real-time updates on equipment status, operation suggestions, and risk alerts. This helps operators intuitively understand the machinery’s working condition and act accordingly. Achieving this requires well-designed voice prompts and trigger mechanisms, along with optimized natural language interaction algorithms to interpret and respond to operator commands. In noisy and complex farmland environments, deep learning can suppress noise to ensure accurate reception and understanding of voice commands. Generative AI can simplify complex instructions into clear language and refine prompts and interfaces based on user feedback. To prevent accidental triggers and command confusion, the system must include well-designed wake words, anti-misactivation mechanisms, and fault-tolerance strategies. These ensure accurate intent recognition, safe operation, and reliable performance of agricultural machinery.

(3) Establish technical standards for the application of intelligent voice systems in agricultural machinery.

The differences in operation methods and communication protocols among agricultural machinery from various manufacturers and models create challenges for the development and application of voice systems. Therefore, establishing unified technical standards can ensure consistent recognition accuracy, response speed, and operational logic in voice control systems for agricultural machinery, improving operational efficiency. Additionally, the establishment of technical standards enhances the safety of agricultural machinery. Unified standards ensure consistent emergency response capabilities and safety measures across different equipment. Moreover, developing technical standards for intelligent voice applications in agricultural machinery can drive the industry’s overall upgrade and transformation. These standards promote communication and collaboration within the agricultural machinery industry, fostering the healthy development of the sector.
